# Polymyxin and lipopeptide antibiotics: membrane-targeting drugs of last resort

**DOI:** 10.1099/mic.0.001136

**Published:** 2022-02-04

**Authors:** Elizabeth V. K. Ledger, Akshay Sabnis, Andrew M. Edwards

**Affiliations:** ^1^​ MRC Centre for Molecular Bacteriology and Infection, Imperial College London, Armstrong Rd, London, SW7 2AZ, UK

**Keywords:** polymyxin, lipopeptide, colistin, daptomycin, antibiotic, resistance

## Abstract

The polymyxin and lipopeptide classes of antibiotics are membrane-targeting drugs of last resort used to treat infections caused by multi-drug-resistant pathogens. Despite similar structures, these two antibiotic classes have distinct modes of action and clinical uses. The polymyxins target lipopolysaccharide in the membranes of most Gram-negative species and are often used to treat infections caused by carbapenem-resistant species such as *Escherichia coli *, *Acinetobacter baumannii* and *Pseudomonas aeruginosa *. By contrast, the lipopeptide daptomycin requires membrane phosphatidylglycerol for activity and is only used to treat infections caused by drug-resistant Gram-positive bacteria such as methicillin-resistant *Staphylococcus aureus* and vancomycin-resistant enterococci. However, despite having distinct targets, both antibiotic classes cause membrane disruption, are potently bactericidal *in vitro* and share similarities in resistance mechanisms. Furthermore, there are concerns about the efficacy of these antibiotics, and there is increasing interest in using both polymyxins and daptomycin in combination therapies to improve patient outcomes. In this review article, we will explore what is known about these distinct but structurally similar classes of antibiotics, discuss recent advances in the field and highlight remaining gaps in our knowledge.

## Polymyxins and lipopeptide antibiotics are drugs of last resort

Currently, daptomycin is the only lipopeptide antibiotic approved for clinical use, whilst two polymyxins, polymyxin B and polymyxin E (known as colistin), are available for human treatment. Daptomycin is considered a last resort antibiotic and so is typically reserved for treating infections where second-line treatments such as vancomycin have failed. It is approved for treatment of complicated skin and soft tissue infections (cSSTIs), bacteraemia and right-sided infective endocarditis caused by methicillin-resistant *Staphylococcus aureus* (MRSA) [1]. As well as this, it is used off-label to treat other infections, including left-sided infective endocarditis, osteomyelitis, septic arthritis, and prosthetic joint infections caused by MRSA and *Enterococcus* species [2, 3]. It is inactive against Gram-negative bacteria and cannot be used to treat lung infections as it is inactivated by the phospholipids in lung surfactant [4].

Whilst clinical trials have shown daptomycin to be safe and efficacious at treating staphylococcal cSSTIs, bacteraemia, endocarditis and osteomyelitis [5–8], there are a significant number of patients in whom daptomycin treatment fails, resulting in poor prognoses [6]. Although there is no consensus on the definition of treatment failure, it is generally considered to be the death of the patient within 30 days of treatment, the presence of persistent infection more than 10 days after the start of treatment or a recurrence of infection within 60 days of the end of treatment [9]. In a study of more than 10 000 patients treated with daptomycin globally between 2004 and 2012, daptomycin showed a clinical success rate of 77 % [6]. However, large variations in success rate were seen depending on the type of infection. For example, daptomycin cured 88 % of uncomplicated SSTIs but only successfully resolved bacteraemia in 70 % of patients [6]. Fortunately, despite early concerns about host toxicity, daptomycin has a similar safety profile to other antibiotics [10].

In contrast to daptomycin, polymyxins are only active against certain Gram-negative bacteria, and are typically only used to treat infections caused by multi-drug resistant organisms including carbapenem-resistant *Enterobacterales *, *Acinetobacter baumannii* and *Pseudomonas aeruginosa *. As the incidence of infections caused by these multi-drug resistant pathogens has increased, so has the use of polymyxins in both high- and low-income settings [11]. Polymyxins are typically given intravenously to treat invasive infections, but there are also preparations for inhalation to treat lung infections, particularly those in people with cystic fibrosis, and it can be given orally to decontaminate the gastrointestinal (GI) tract [12].

Unfortunately, polymyxins are not particularly effective and fail to eradicate the causative pathogen in up to of 70 % infections [13]. Furthermore, polymyxin therapy is frequently associated with host toxicity, particularly nephrotoxicity and to a lesser extent neurotoxicity [14, 15]. Toxicity appears to be dose-dependent and the high frequency of nephrotoxicity is explained by pharmacokinetic studies showing that polymyxins concentrate in the kidneys [16, 17]. Several underlying mechanisms have been proposed to be responsible for host toxicity, including the presence of d-amino acids in polymyxins and disruption of host cell membrane function [14, 15].

This host toxicity greatly complicates treatment and prevents the use of higher doses to improve treatment outcomes [18]. This is important because polymyxin antibiotics are thought to achieve a sufficient serum concentration to kill bacteria in only 50 % of patients [19, 20]. Although newer agents have been licensed to combat drug-resistant Gram-negative pathogens, these are not available in all parts of the world and thus it is likely that polymyxins will continue to be used as a last resort for some time to come [21].

Therefore, based on the clinical picture and the lack of alternatives in many parts of the world, new approaches are desperately needed to improve the efficacy of polymyxins and lipopeptide antibiotics. This requires fundamental studies to determine their mechanisms of action, as well as clinical studies of combination therapies and the development of next generation members of these classes of antibiotics.

## Polymyxins and lipopeptide antibiotics are structurally similar

Daptomycin was discovered in 1983, making it one of the most recently discovered new classes of antibiotic to enter clinical use. It was discovered during characterization of a strain of the bacterium *Streptomyces roseosporus* isolated from a soil sample taken from Mount Ararat in Turkey and was approved for human use in 2003 after a lengthy and eventful development process that required the development of a dosing regimen that significantly reduced host toxicity [22]. The optimal dosing strategy was counter-intuitive because it found that a single large daily dose was considerably less toxic than three smaller doses per day but equally effective in treating infection [18].

**Fig. 1.**
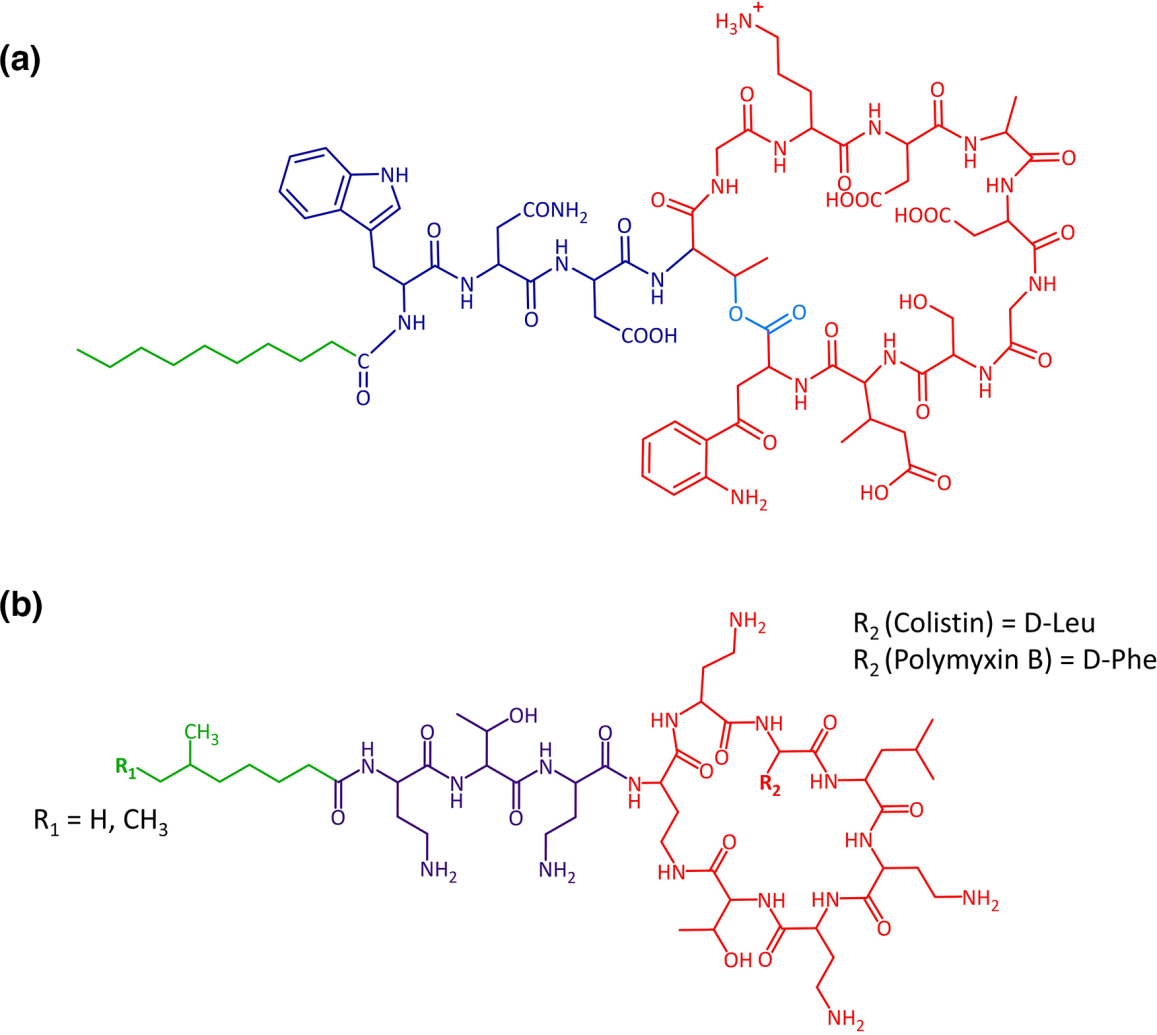
Structures of daptomycin and polymyxin B/colistin. The structure of daptomycin is shown in (**a**) and the structure of polymyxin B/colistin in (**b**), with differences between the two polymyxins indicated. In both cases, the peptide ring is shown in red, the exocyclic tripeptide in purple and the lipid tail in green, while the ester bond of daptomycin is shown in light blue.

Daptomycin is a cyclic lipopeptide, composed of a 13-residue peptide and a decanoyl fatty acid moiety (Fig. 1a) [23]. This antibiotic is synthesized non-ribosomally and contains non-proteinogenic amino acids, including d-enantiomers, ornithine, (2*S*,3*R*)-methylglutamate and kynurenine [23]. The C-terminal ten residues of the peptide are linked by an ester bond to form a macrocyclic core while the N-terminal three residues are not part of the ring but join the ring to the decanoyl fatty acyl residue [23]. Daptomycin is anionic, but acquires a cationic charge in the presence of calcium ions, which are essential for antibacterial activity [24].

In contrast to daptomycin, the polymyxins were one of the earliest classes of antibiotics to be discovered, with polymyxin B first described in 1947 and polymyxin E (colistin) identified in 1949, when it was isolated as a secondary metabolite from a flask of fermenting *Paenibacillus polymyxa* var. *colistinus* [25–27]. As is the case for daptomycin, colistin and polymyxin B are produced by non-ribosomal peptide synthetase systems [26, 27].

Colistin and polymyxin B are structurally very similar, consisting of cyclic lipopeptide compounds composed of 10 amino acids, arranged as a circular heptapeptide linked to an exocyclic tripeptide, which in turn is attached to a fatty acid residue (Fig. 1b) [28]. Polymyxin B and colistin differ only by a single amino acid residue at position 6 of the antibiotics’ chemical structures. Colistin has a d-leucine group at this position, whereas polymyxin B contains a d-phenylalanine isomer [28]. The other nine amino acids of polymyxins are a variety of d-leucine and l-threonine residues, as well as five conserved l-α-γ-diaminobutyric acid (DAB) residues at positions 1, 3, 5, 8 and 9 of the antibiotic molecules. These DAB residues are crucial for conferring the heptapeptide ring in the C terminus of polymyxin compounds with a net positive charge at physiological pH, and the cationic, hydrophilic nature of this macrocycle is essential for colistin’s antimicrobial properties, as is the lipid tail [28, 29].

## Polymyxins and lipopeptide antibiotics have completely different spectra of activity and their mechanisms of activity are poorly understood

Both daptomycin and the polymyxins cause membrane damage to their bacterial targets and are rapidly bactericidal *in vitro*. However, there is debate regarding the mode of action of both classes of antibiotic, and significant gaps in our understanding of how they kill bacteria remain. This is important because a better understanding of the mechanism of action may identify improved ways of using these drugs, as well as support efforts to generate improved polymyxin and lipopeptide antibiotics. Furthermore, a comprehensive understanding of how lipopeptide and polymyxin antibiotics function would contribute to the growing interest in developing new antibiotics that inhibit the synthesis or transport of membrane components [30]. We summarize here what is known and areas of controversy.

## Polymyxins

Polymyxins have a high affinity and specificity for lipopolysaccharide (LPS) over other membrane components such as phospholipids [31]. In particular, the primary targets of colistin are the negatively charged phosphate groups within the lipid A domain of LPS present in both the outer- and cytoplasmic membranes, explaining why polymyxin compounds only possess antimicrobial properties against Gram-negative strains [28, 32]. The amphipathic nature of colistin’s chemical structure is critical to its function, with three-dimensional NMR experiments revealing that the binding of the antibiotic to LPS is mediated by electrostatic interactions between the positively charged DAB residues in the polymyxin’s heptapeptide macrocycle and the anionic lipid A moiety [33].

The arrangement of LPS molecules in the outer membrane of Gram-negative bacteria is stabilized by divalent cations, in particular Mg^2+^ and Ca^2+^, which form electrostatic bridges between individual lipid A domains in the outermost leaflet of the cell surface bilayer. Colistin competitively displaces cations away from lipid A, resulting in outer membrane destabilization. Crucially, it has been demonstrated that the colistin-induced displacement of Mg^2+^ and Ca^2+^ ions is not dependent on the antibiotic’s entry into the cell, and that this process can be inhibited when these cations are present extracellularly in excess [31, 34].

Whilst this primary interaction between colistin and LPS is well established and has been extensively studied and characterized, the subsequent stages in the mechanism of bactericidal activity are very poorly understood. It is hypothesized that colistin traverses the compromised outer membrane via a process termed ‘self-directed uptake’ [28, 35, 36]. In this process, the destabilizing and weakening of the outer membrane through binding of the antibiotic’s positively charged peptide ring with LPS creates space that enables colistin to insert its N-terminal fatty acyl chain into the outermost leaflet of the outer membrane [28, 35, 36]. This lipophilic tail of the polymyxin structure then interacts with the hydrophobic fatty acid residues that comprise the inner portion of the LPS lipid A domain, resulting in further damage to the cell surface bilayer [28, 35, 36]. Whilst this model fits much of the available data, there is very limited direct evidence for self-directed uptake.

Permeabilization of the outer bacterial membrane provides colistin molecules with access to the cytoplasmic membrane, the disruption of which is crucial for bactericidal activity [37, 38]. However, whilst polymyxin-mediated membrane disruption can cause lysis, this is not required for bacterial killing [37, 38].

**Fig. 2.**
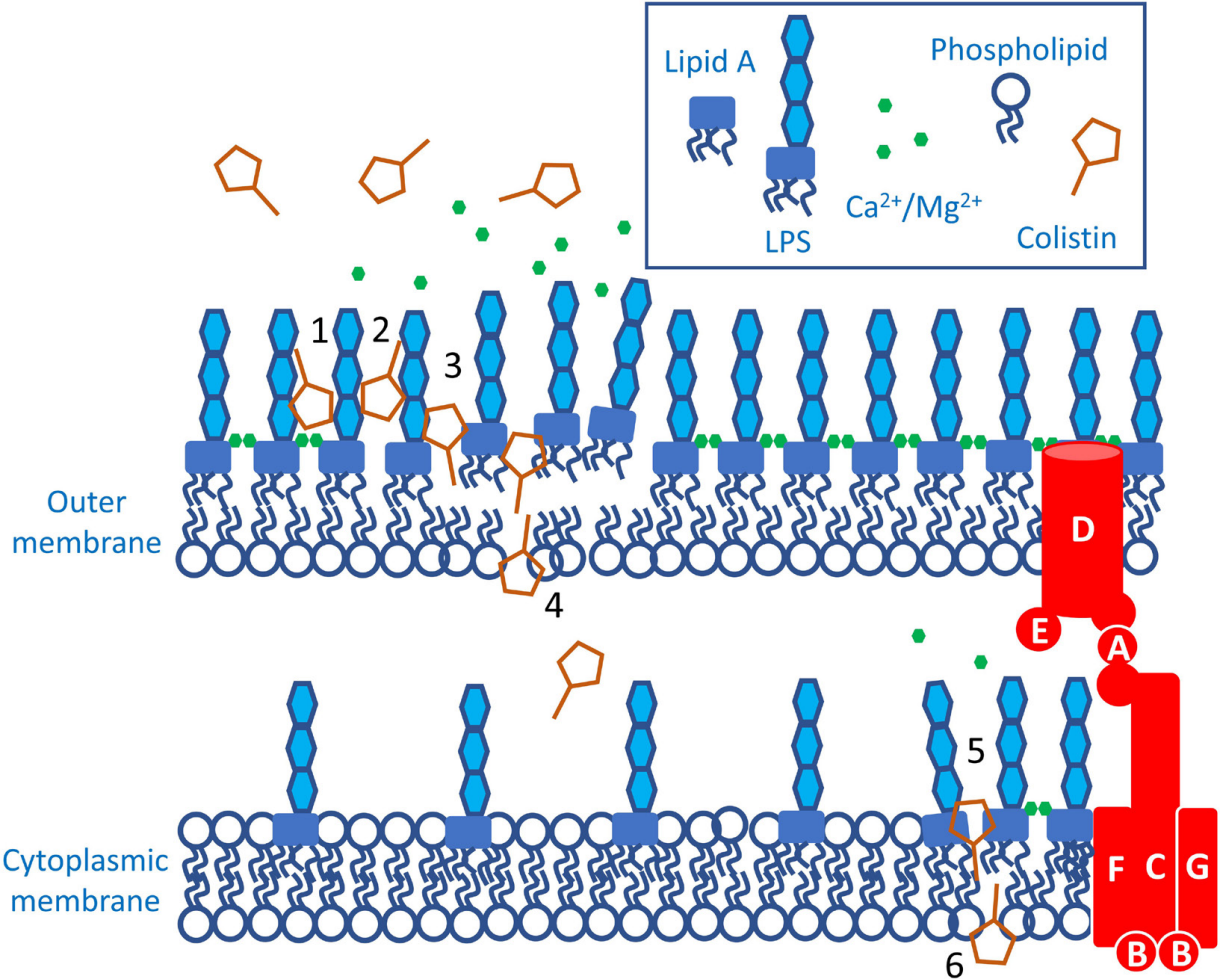
Current model for the mechanism of action of polymyxin antibiotics. The cationic peptide ring of the polymyxin (colistin) interacts with lipid A of LPS (1), leading to displacement of the cation bridges and weakening of the outer leaflet of the outer membrane (OM) (2). The lipid tail of colistin inserts into the outer leaflet, further weakening the OM (3). The polymyxin then traverses the OM via self-promoted uptake and enters the periplasm (4). The polymyxin then engages LPS in the cytoplasmic membrane (5) before it is transported to the OM by the multi-component Lpt system (in red), leading to disruption of this structure, escape of cytoplasmic contents, and possibly downstream effects such as production of reactive oxygen species, followed by bacterial death and lysis (6).

Disruption of the cytoplasmic membrane requires the polymyxin lipid tail, which was hypothesized to enable insertion of the polymyxin into the phospholipid bilayer [28, 35, 36]. However, recent work from our group has shown that colistin-mediated disruption of the cytoplasmic membrane is dependent upon polymyxin targeting of LPS as it is being trafficked to the outer membrane [37] (Fig. 2). When the abundance of LPS in the cytoplasmic membrane was increased via pharmacological means, bacteria became much more susceptible to killing by colistin [37]. By contrast, when LPS in the cytoplasmic membrane was chemically modified to reduce its affinity for colistin, bacteria and spheroplasts were protected from colistin-mediated disruption but not damage caused by other cationic antibacterial peptides [37] (Fig. 2). This finding explains how an antibiotic with high selectivity for LPS over phospholipids can disrupt both membranes, and is supported by studies with synthetic membranes showing that LPS was necessary for colistin-mediated permeabilization, even when it was present at only 3 % of the total membrane composition [31]. Furthermore, it agrees with the finding that some strains of *A. baumannii* can acquire resistance to very high concentrations of colistin via loss of LPS biosynthesis, resulting in outer and cytoplasmic membranes that consist of phospholipid bilayers [39]. Polymyxin-mediated disruption of the cytoplasmic membrane is sufficient to allow ingress of the fluorescent dye propidium iodide [37, 38] and the egress of small molecules such as potassium ions, amino acids and uracil, as well as proteins such as beta-galactosidase [40, 41]. However, it is not clear to what extent the release of these molecules is due to the initial interaction of polymyxins with LPS or the subsequent lysis. This is because we have no understanding of how the interaction of polymyxins with LPS in the cytoplasmic membrane results in membrane permeabilization leading to bacterial killing. It is possible that polymyxins directly permeabilize the cytoplasmic membrane, as observed with synthetic membranes [31], but the antibiotic may conceivably act via another mechanism, such as by inhibiting LPS transport to the outer membrane.

Whilst the prevailing model for polymyxin’s mode of action is sequential outer and cytoplasmic membrane disruption as described above, other models have been proposed. One of these alternative models is known as the ‘vesicle–vesicle contact pathway’ [42]. In this model, polymyxins present in the inner leaflet of the outer membrane bind to anionic phospholipids in the cytoplasmic membrane, generating stable contacts. This leads to the rapid exchange of phospholipids between the two bilayers and ultimately lytic cell death [43]. However, this model of colistin-mediated killing of bacteria remains largely putative, with a lack of experimental evidence beyond studies with synthetic membrane vesicles, and it is not clear how membrane contact could occur in bacterial cells given the presence of the cell wall.

Whilst there is overwhelming evidence that polymyxins cause membrane disruption [23, 32, 33], additional mechanisms have been proposed to contribute to bacterial killing. These have been summarized in detail elsewhere [44] and so only a brief overview is provided here. Colistin, and other polymyxins, have been found to directly inhibit the enzymatic activity of the respiratory enzyme NDH-2 from *E. coli *, *K. pneumoniae* and *A. baumannii* [45]. However, the concentrations of antibiotic required for 50 % inhibition of NDH-2 are >50 fold higher than the typical MIC (1 µg ml^−1^) and this model remains to be fully tested in a whole-cell model [45]. However, it may be that disruption of the cytoplasmic membrane indirectly affects the respiratory complex, leading to synergistic inhibitory activity.

Similarly to several other antibiotic classes, colistin has been shown to mediate cell death of *A. baumannii* through formation of reactive oxygen species as part of the ‘hydroxyl radical death pathway” [46], although this was not replicated in a similar study with *P. aeruginosa* [47].

It has also been suggested that polymyxins may target the A-site of prokaryotic 16S rRNA, the cognate target of aminoglycoside antibiotics [48]. However, colistin does not block bacterial translation, and the authors of this work concluded that it is unlikely that polymyxin drugs kill cells via an aminoglycoside-like mechanism of action [48].

## Daptomycin

As for the polymyxins, the mode of action of daptomycin has been the subject of several studies, leading to different conclusions about its mode of action [23, 24, 49–55]. However, it is clear that the lipopeptide antibiotic binds to phosphatidylglycerol (PG) in the bacterial membrane and requires the presence of this phospholipid for activity [56, 57]. It therefore shows much lower activity against Gram-negative and eukaryotic membranes, which have much lower PG contents than those of Gram-positive bacteria [58, 59].

**Fig. 3.**
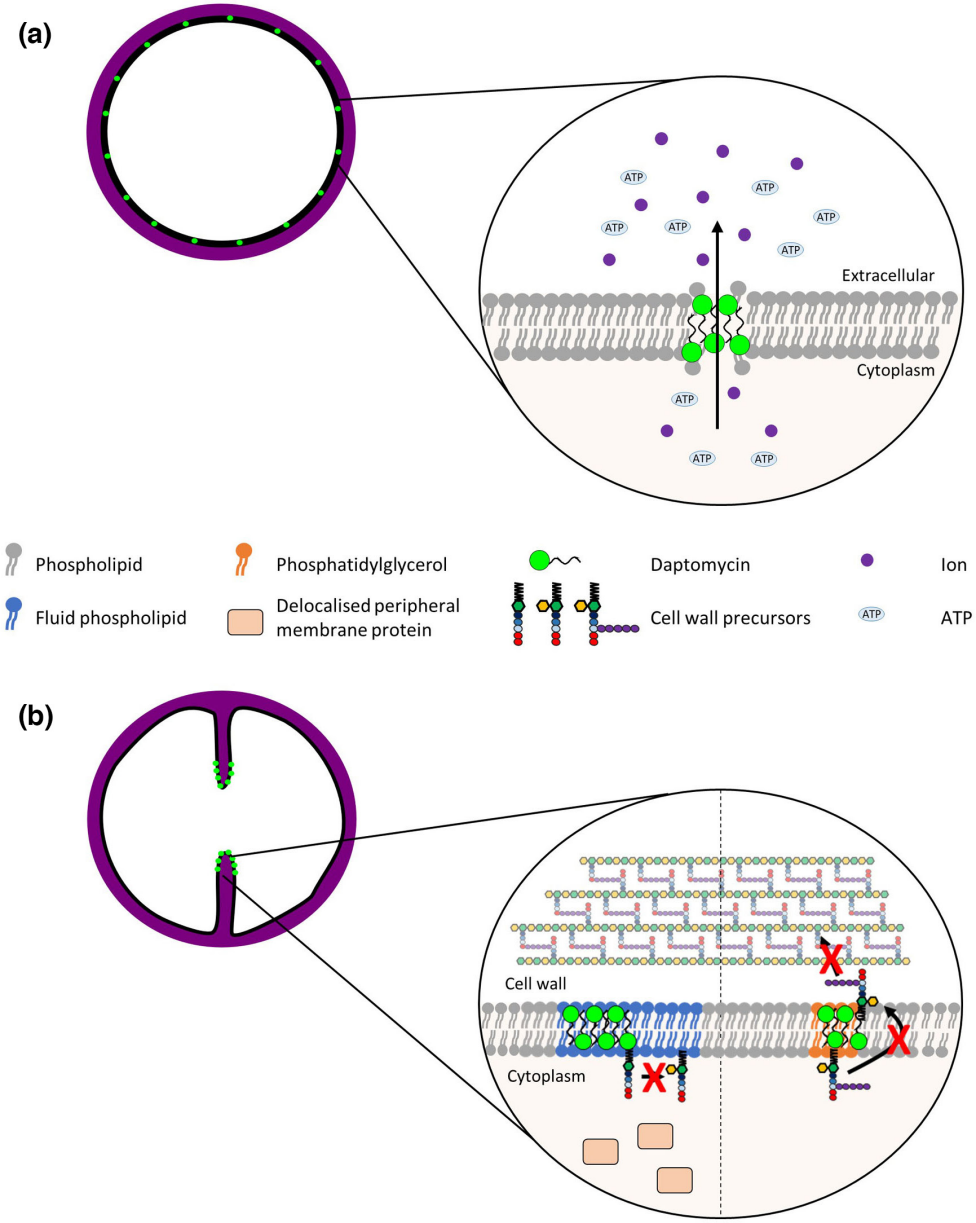
Proposed mechanisms by which daptomycin disrupts membrane integrity and cell wall biosynthesis. Daptomycin disrupts the membrane by forming oligomeric complexes with phosphatidylglycerol, leading to leakage of ions and ATP out of the cell (**a**). Daptomycin binding at division septa leads to inhibition of cell wall synthesis via two proposed mechanisms (**b**). In the first model, daptomycin binds to fluid regions of the membrane, causing membrane rigidification. This leads to the mislocalization of peripheral membrane proteins involved in peptidoglycan synthesis to the cytoplasm, decreasing cell wall synthesis. In the second model, daptomycin binds to a complex consisting of phosphatidylglycerol, calcium ions and membrane-bound cell wall precursors, inhibiting the translocation of peptidoglycan precursors across the membrane. These two mechanisms are not mutually exclusive and may act synergistically to inhibit cell wall biosynthesis.

Daptomycin has a net negative charge of −3 at physiological pH, and so it must bind calcium ions to interact with the anionic PG [24, 52, 56, 60]. Due to its net positive charge in the presence of calcium, and its ability to interact with membranes, daptomycin is primarily considered to have a membrane-disrupting mode of action similar to that of antimicrobial peptides (AMPs) [24]. In support of this, daptomycin has been shown to oligomerize in a calcium-dependent manner, before binding to PG and inserting into the bacterial membrane (Fig. 3a) [51, 57]. This disrupts the integrity of the membrane, possibly via the formation of pores, and leads to the loss of intracellular ions and ATP [24, 61, 62]. Depletion of cellular ATP affects macromolecular synthesis, reducing peptidoglycan, DNA, RNA and protein synthesis and resulting in cell death [53, 63].

However, as well as compromising membrane integrity, insertion of daptomycin into the membrane is also thought to directly disrupt peptidoglycan synthesis (Fig. 3b) [54, 55, 64]. In support of this, analysis of bacteria exposed to daptomycin showed upregulation of genes involved not only in membrane stress but also in cell wall stress [54, 65]. Furthermore, in actively dividing cells, daptomycin has been observed to bind preferentially to the fluid membrane microdomains that contain enzymes required for cell wall synthesis [54, 55], and the binding of daptomycin to these regions results in membrane rigidification [54]. As the fluidity of these regions is essential for the correct localization of several peripheral membrane proteins involved in peptidoglycan synthesis, the increase in membrane rigidity at these sites may therefore lead to delocalization of these essential proteins, inhibiting peptidoglycan synthesis [54, 55].

An additional mechanism (or extension of the mechanism above) by which daptomycin may inhibit cell wall synthesis has also been proposed (Fig. 3b) [64]. In this model, daptomycin functions in a similar way to other cyclic lipopeptides, such as amphomycin and fruilimicin, which both inhibit cell wall synthesis by sequestering lipid-bound peptidoglycan precursors [64]. In support of this, daptomycin has been shown to activate VraSR, a two-component regulatory system activated by antibiotics which interfere with the lipid II cycle controlling the cell wall stress response [65, 66]. Grein *et al.* proposed that after binding to the membrane at the septum of *S. aureus *, daptomycin interacts with bactoprenol-bound peptidoglycan precursors in a calcium- and PG-dependent manner [64]. This disrupts peptidoglycan synthesis and correlates with a rapid loss in bacterial viability [64]. After initial binding at the septum of *S. aureus *, daptomycin then disperses through the membrane, leading to widespread membrane depolarization and a loss of membrane integrity [64]. Therefore, daptomycin-mediated disruption of fluid microdomains may compromise cell wall synthesis by disrupting the function and/or localization of synthetic enzymes and by sequestering lipid II, which is enriched at these sites [67, 68].

However, studies using isothermal titration calorimetry and synthetic membrane vesicles did not identify significant binding of lipid II by daptomycin in the presence of PG, in contrast to that seen with nisin or teixobactin [69]. Furthermore, whilst purified PG blocks the bactericidal activity of daptomycin [70], lipid II does not [71]. Therefore, the role of lipid II in the mechanism of action of daptomycin is unclear. It is also important to note that daptomycin may act slightly differently depending on the bacterial species examined, which are principally *S. aureus* and *Bacillus subtilis *.

Taken together, whilst there is strong evidence that daptomycin targets phosphatidylglycerol in the bacterial membrane, the mechanism(s) by which this leads to bacterial killing is unclear. Whilst daptomycin is able to disrupt model membranes and kill both cell wall-lacking protoplasts and cells of *S. aureus* which are not actively dividing and lack a septum [57, 72, 73], this does not rule out that inhibition of cell wall synthesis contributes to killing of *S. aureus *.

## Resistance to, and tolerance of, polymyxin and lipopeptide antibiotics involves modifications to the cell envelope

The incidence of polymyxin resistance in human pathogens is relatively rare in wealthy nations, but is often more common in low- and middle-income countries. For example, 1.8 % of *A. baumannii* in Europe were found to be colistin-resistant compared with 6.7 % of isolates in South East Asia [74]. Similarly, a recent study of carbapenem-resistant *Enterobacterales* (CRE) in the USA found 7.1 % were resistant and 10.1 % were heteroresistant [75]. By contrast, up to 27.1 % of CRE in Brazil were reported to be polymyxin-resistant in 2016, which marked a rapid increase from 2011 when no polymyxin isolates were identified [76, 77]. It is important to note that polymyxins have been used as growth promoters and the incidence of resistance to these antibiotics is therefore relatively common in bacteria from livestock, which may be transferred to human pathogens in the case of mobile colistin resistance genes (see below) [78].

Since these drugs are used as last resorts, resistance can have very serious adverse impacts on patient outcomes. In a recent study of CRE bloodstream infection, 28 day mortality was 39 % when isolates were colistin-susceptible and 51 % in the case of polymyxin strains [79]. This is in keeping with previous work showing significantly higher mortality when infections are caused by polymyxin-resistant strains [80].

Daptomycin resistance is rare globally, reflecting the fact that this drug is reserved as a last resort in humans, is rarely used in low- or middle-income settings and is not used for livestock [81, 82]. However, daptomycin resistance can emerge during treatment, and reduced susceptibility is often seen in vancomycin-insensitive strains [83]. Daptomycin treatment is therefore more likely to fail when the causative organism is resistant to vancomycin [84].

As the use of polymyxins and daptomycin increases, it is likely that the frequency of resistance will increase [76, 83]. However, monitoring the incidence of resistance is challenging because the definitions of polymyxin and lipopeptide resistance are the subject of intense debate [20, 85]. A further complication is that accurate polymyxin susceptibility testing can be difficult to achieve because of the poor penetration of these antibiotics into agar and their propensity to bind to plastic surfaces [86]. These and additional issues related to polymyxin susceptibility testing have been reviewed in depth recently elsewhere [20, 86].

## Intrinsic polymyxin resistance

While polymyxin antibiotics possess antimicrobial activity against most Gram-negative pathogens, several species are intrinsically resistant, including strains of the *Burkholderia cepacia* complex, *Proteus mirabilis* and bacteria in the genus *Serratia* [87, 88]. In all these species, intrinsic colistin resistance is due to the synthesis of LPS that is modified by the addition of cationic chemical groups consisting of phosphoethanolamine (PEtN) and/or 4-amino-4-deoxy-l-arabinose (l-Ara4N) [30, 88]. Both PEtN and l-Ara4N moieties are added to the lipid A domain of LPS during its transit to the outer membrane, and their cationic properties decrease the net negative charge of lipid A conferred by its phosphate groups, which reduces affinity for the cationic polymyxins [30, 89]. Only a single bacterial species, *B. polymyxa* var. *colistinus*, which synthesizes colistin, is known to be intrinsically resistant via the production of a hydrolytic enzyme that degrades the polymyxin antibiotic [90].

## Polymyxin resistance acquired via mutation

Modification of LPS with l-Ara4N requires products of the *arnBCADTEF* operon, whilst PEtN addition requires the *eptA*/*eptB* genes encoding PEtN transferases [30, 89]. Whilst similar systems are also found in many polymyxin-susceptible bacteria, they are not constitutively active [30, 89]. However, polymyxin resistance is often acquired in several different species via gain-of-function mutations in genes encoding regulatory systems such as PhoPQ, MgrB or PmrAB (BasRS) that regulate expression of the *arn* operon and *eptA*/*eptB*, leading to constitutive expression and thus polymyxin resistance [91–95].

In isolates of *P. aeruginosa *, mutations in the genes encoding several additional two-component systems have been implicated in polymyxin resistance, including ParRS, CprRS and ColRS [96, 97]. It is thought that all of these complexes are linked to expression of the *arn* operon, leading to an increase in the lipid A-modifying activity of the proteins produced from the operon [96, 97]. However, the precise molecular signalling pathway(s) by which this occurs remain(s) poorly characterized.

Uniquely, some strains of *A. baumannii* have acquired polymyxin resistance via the complete loss of LPS due to spontaneous mutations or insertion of the transposable element *ISAba11* into genes responsible for LPS biosynthesis, including *lpxA*, *lpxC* and *lpxD* [39, 98]. This mechanism of resistance is restricted to strains of *A. baumannii* deficient for PBP1a expression, and cannot occur in most other Gram-negative bacteria because the loss of LPS biosynthesis is typically lethal [30, 89, 99].

## Polymyxin resistance due to efflux

As well as mutations that mediate resistance to polymyxins through changes to LPS, mutations that cause increased production of the MexXY-OprM efflux pump complex in *P. aeruginosa* have also been linked to decreased colistin susceptibility [100]. Similarly, mutations resulting in over-expression of *oprH*, which encodes the small outer membrane protein OprH, lead to a reduction in susceptibility to colistin [101], whilst the efflux pumps KpnEF and AcrAB have also been implicated in polymyxin resistance [102, 103]. Further evidence for efflux of colistin is provided by studies showing that an inhibitor of multi-drug efflux pumps increased susceptibility to colistin in several strains of bacteria, including intrinsically-resistant organisms [104–106].

However, it has yet to be directly demonstrated that colistin is a substrate for efflux systems, and polymyxins are significantly larger than previously described antibiotic substrates [104–107].

## Polymyxin resistance via acquired genes

In addition to spontaneous resistance due to genetic changes and efflux pumps, a worrying development in recent years is the emergence of plasmids conferring polymyxin resistance via the presence of mobile colistin resistance (*mcr*) genes [108]. Since this has been summarized in detail recently, we focus here on the key points [108, 109].

The first *mcr* gene was identified in 2015 on an IncI plasmid from a strain of *E. coli* obtained in China from farming animals, and termed *mcr-1* [110]. However, it is believed that the gene had been in existence and circulating worldwide for several decades beforehand [111], with the resistance determinant found in more than 40 countries across five continents, and in a diverse range of *Enterobacteriaceae* strains (* E. coli *, *K. pneumoniae*, *S. enterica*, *Shigella sonnei *, *Enterobacter* species, *Citrobacter* species and *Moraxella* species) [108]. Although initially detected as being harboured on an IncI plasmid, it has been shown that carriage of the *mcr-1* gene in nature can occur on various plasmid types, including IncF, IncHI2, IncP, IncX4 and IncY [109]. Furthermore, the reservoirs of *mcr-1*-bearing plasmids are similarly wide-ranging, with these mobile genetic elements identified in food (meat/vegetables), aquatic environments, hospital sewage, infected/colonized humans, wild birds/animals and especially predominantly in farm animals [112–114]. It is hypothesized that livestock, particularly cattle and pigs, are a major source and driver of global *mcr-1* spread, and the extensive use of colistin in veterinary medicine as a growth promoter has only served to accelerate and exacerbate this problem [115]. In support of this, bans on administration of polymyxin antibiotics to farm animals has led to a drastic reduction in colistin resistance being detected in both livestock and human patients in several countries [115].

The gene product of *mcr-1* (MCR-1) is a lipid A-modifying phosphoethanolamine transferase enzyme that is a member of the YhjW/YjdB/YijP alkaline phosphatase protein superfamily and functions by adding a positively charged PEtN group to the negatively charged phosphate groups of lipid A, as occurs in intrinsic resistance or in that acquired via spontaneous mutation [110].

Based on *in silico* analyses, the *mcr-1* gene is hypothesized to have originated from an intrinsically polymyxin-resistant Gram-negative bacterium such as *Moraxella *, *Vibrio *, *Limnobacter *, *Enhydrobacter* or *Methylophilaceae* [116, 117]. Moreover, structural studies of the MCR-1 protein have indicated significant similarity to two additional PEtN transferase enzymes from intrinsically resistant strains: EptC from *Campylobacter jejuni *, and LptA from *Neisseria meningitidis* [118].

To date, 10 *mcr* homologues have been described [108]. However, although all 10 encode PEtN transferase enzymes, the similarity in their amino acid identity compared with *mcr-1* can vary substantially, indicating that they may have originated from disparate genetic sources [108]. The impact of these differences on colistin resistance is not well understood. However, recent work from our laboratory indicates there may be subtle differences in the susceptibility of strains with different MCR types to colistin-mediated membrane damage [38].

Uniquely and surprisingly, it has been found in *S. enterica* clinical isolates that the *mcr-9* gene does not appear to confer polymyxin resistance [119]. Expression of the *mcr-9* gene via an inducible promoter confirmed that this gene encodes a PEtN transfer capable of modifying lipid A, but the reason why *mcr-9* does not confer colistin resistance in clinical isolates grown *in vitro* is unclear [119, 120]. It is possible that *mcr-9* may only be expressed under certain environmental conditions not present *in vitro*, including in response to colistin, but this has not been tested fully and most strains lack the regulatory system proposed to induce *mcr-9* in response to colistin [120–122]. Despite not conferring colistin resistance, the *mcr-9* gene is one of the most widely disseminated *mcr* homologues detected in clinical isolates, which may suggest an alternative function [108].

In addition to *mcr* genes, recent analysis of a bovine *E. coli* isolate from 2015 identified an IncFII plasmid containing a copy of the *arnBCADTEF* operon that was closely related to the *arn* operon from *Kluyvera ascorbate* [123]. This element (termed pArnT1) was subsequently demonstrated to mediate colistin resistance, confirming an additional mechanism of mobilized colistin resistance [123]. However, it remains to be determined how well disseminated this element is.

Although LPS modified with l-Ara4N and/or PEtN is present at both the outer and cytoplasmic membranes of bacteria with acquired polymyxin resistance, this does not protect both membranes equally [37, 38]. Bacteria with resistance via spontaneous mutation or acquisition of an *mcr* gene are still susceptible to outer membrane damage, as assessed using ingress of fluorescent dyes and antibiotics [37, 38]. By contrast, the cytoplasmic membranes of colistin-resistant bacteria are resistant to damage, enabling bacterial survival [37, 38]. This is thought to be due to the presence of large concentrations of unmodified LPS in the outer membrane, whereas the abundance of LPS in the cytoplasmic membrane is so low that even if only a fraction of molecules are modified it is sufficient to confer protection [37, 38]. This may have important implications for treatment, since colistin-mediated permeabilization of the outer membrane of polymyxin-resistant bacteria sensitizes the organism to antibiotics such as rifampicin [38, 87, 124].

## Polymyxin heteroresistance

Heteroresistance refers to the presence of a drug-resistant subpopulation of bacteria within a larger population of antibiotic-susceptible bacteria [75, 125, 126]. This contrasts with conventional resistance where all or most members of the population are resistant to a particular drug. The heteroresistance phenotype is often under-appreciated since it can be challenging to identify using conventional diagnostic approaches. Whilst it may manifest as ‘skipped wells’ in broth microdilution testing, this is not always the case and up to half of colistin heteroresistant isolates are classified as susceptible [75].

Recent work from the USA has undertaken a large, systematic analysis of colistin heteroresistance in CRE between 2012 and 2015 [75]. This revealed that the incidence of heteroresistance (10.1 % of isolates) was greater than that of conventional resistance (7.1%). As such, colistin heteroresistance is a potentially very significant threat. However, it is not yet clear what the clinical impact of heteroresistance is on patient outcomes. Studies in mice indicate that heteroresistance can cause colistin treatment failure, but it is not yet clear if this is the case in humans, not least because of the challenges in identifying heteroresistance [126–128]. It is also unclear whether colistin heteroresistance is a precursor to conventional resistance, as appears to be the case for beta-lactam resistance [126–129].

The molecular basis of heteroresistance appears to involve mutations in the same genes as conventional colistin resistance [130], but the precise factors that determine whether a specific member of the population is susceptible or resistant remain to be determined [125].

## Daptomycin non-susceptibility and resistance

For *Enterococcus* species, the daptomycin breakpoint has been revised recently and remains the subject of debate [131, 132]. As such, the definition of resistance has changed over time and several different mechanisms have been proposed to mediate resistance [133].

For staphylococcal strains, a breakpoint has not been defined and so strains with an MIC of >1 μg ml^−1^ are classed as daptomycin non-susceptible (DNS), rather than resistant [23, 134]. Increases in MIC are often small, but these are clinically important as there is limited scope for increasing the daptomycin dose due to toxicity [135–137].

A dedicated resistance mechanism has not been identified in either staphylococci or enterococci but instead a variety of changes to the cell envelope have been associated with reduced daptomycin susceptibility [134]. These changes depend on the organism, with altered membrane composition thought to be important for mediating resistance in enterococci, while changes to both the cell membrane and cell wall can confer the DNS phenotype in *S. aureus.*

## Non-susceptibility due to changes in membrane composition

**Fig. 4.**
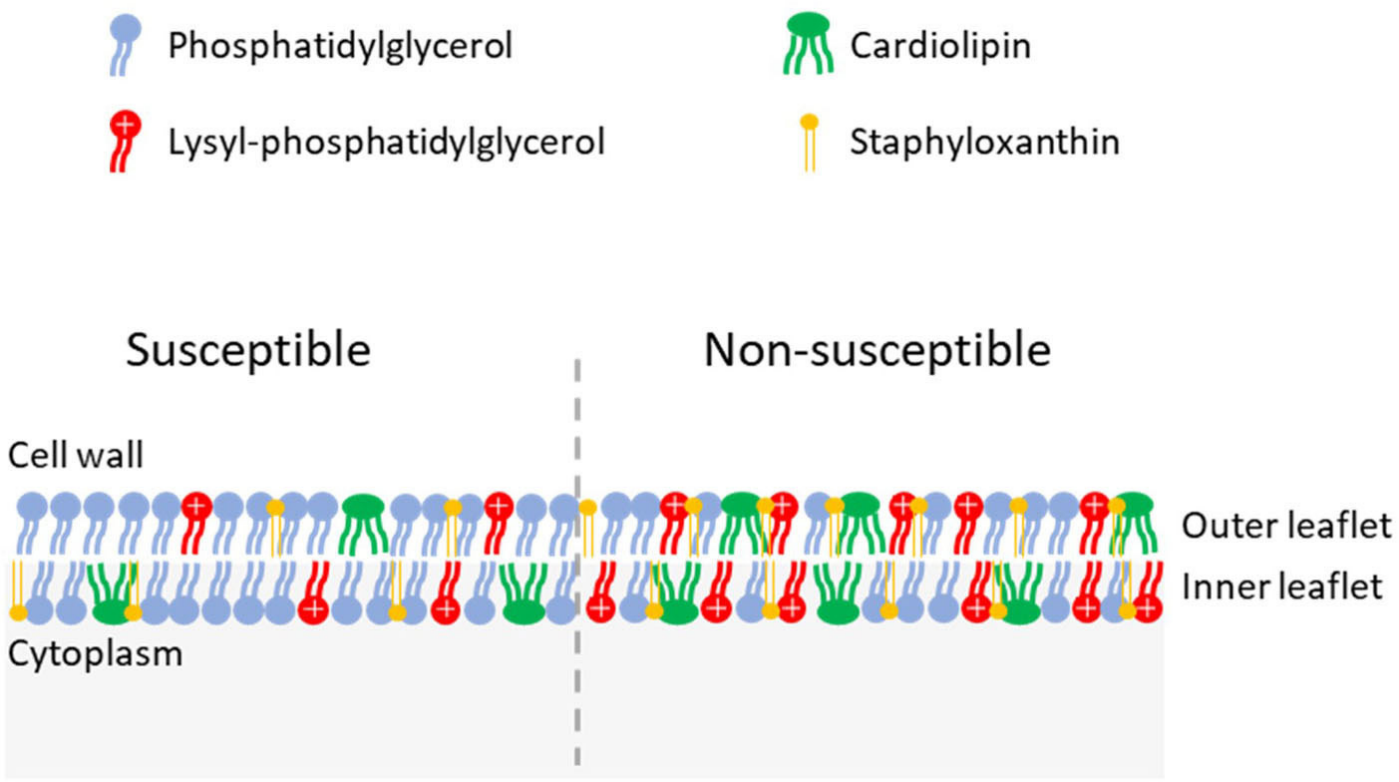
Cell membrane of daptomycin-susceptible (left) and non-susceptible (right) *S. aureus *. Daptomycin non-susceptible strains have been observed to have decreased phosphatidylglycerol and increased cardiolipin, lysylphosphatidylglycerol and staphyloxanthin compared to susceptible strains.

*S. aureus* DNS isolates and vancomycin-resistant enterococci (VRE) daptomycin-resistant isolates often have altered membrane composition compared to susceptible strains (Fig. 4). Since daptomycin requires PG for its bactericidal activity, one important difference between the membrane compositions of susceptible and non-susceptible strains is the relative abundance of this lipid [138–141]. The elimination of PG from the membrane results in extremely high daptomycin MICs (>256 μg ml^−1^) [142, 143]. However, while this has been observed in some Gram-positive species, including *Corynebacterium striatum* and *Streptococcus mitis */*oralis,* this has not been observed in *S. aureus* or enterococci*,* which require PG for viability [142, 143]. Instead, reduced susceptibility is associated with decreased PG content, in the case of *S. aureus* via mutations in the PG synthase, *pgsA* [138].

As PG is a precursor for both lysylphosphatidylglycerol (LPG) and cardiolipin (CL), another mechanism to decrease the PG content is increased synthesis of either of these species. LPG is synthesized by MprF, and SNPs in the gene encoding MprF are the most frequent genetic changes associated with DNS [138, 144–146]. These SNPs are typically gain-of-function mutations, leading to increased rates of LPG synthesis or translocation to the outer leaflet of the membrane [139, 144, 147, 148]. As well as protecting bacteria from daptomycin by decreasing membrane PG content, the positive charge of LPG has been hypothesized to lead to repulsion of the positively charged lipopeptide through electrostatic interactions [134, 139, 148, 149]. However, several SNPs have been identified at the junction of the synthase and flippase domains of *mprF,* which do not affect LPG synthesis or translocation or membrane surface charge [144]. Instead, it has been hypothesized that these SNPs may alter the substrate affinity of the flippase domain, enabling MprF to translocate daptomycin, resulting in reduced susceptibility [144]. Membrane LPG content also affects fluidity, which in turn may mediate reduced daptomycin susceptibility (see below) [150].

Since two molecules of PG are needed to synthesize one molecule of CL, increases in CL can also lead to decreases in PG [151–153]. In line with this, SNPs in the gene encoding one of the CL synthases of *S. aureus, cls2,* have been identified in DNS isolates [138, 152]. These SNPs resulted in an increase in the membrane composition of CL and a decrease in PG. High concentrations of CL have been demonstrated to inhibit the ability of daptomycin to permeabilize model membranes, while the SNPs in *cls2* led to an increase in the thickness of the membrane, reducing the ability of daptomycin to permeabilize the membrane [152, 154]. Similarly, SNPs in the cardiolipin synthase gene *cls* of *Enterococcus* species have been linked to daptomycin resistance by increasing CL content and thereby decreasing PG abundance [140, 155]. In addition to decreasing PG content, it has been proposed that CL helps divert daptomycin away from the division septum, reducing its damaging effects on the cell [140]. *Enterococcus* can also acquire daptomycin resistance via gain-of-function mutations in the genes encoding stress response systems, particularly LiaFSR/LiaSR [141, 156]. This confers decreased susceptibility to daptomycin via diverse routes, including changes in membrane lipid composition, depending on the environment [157].

Finally, studies have identified that changes in the fluidity of the cell membrane are associated with altered daptomycin susceptibility in both *S. aureus* and *Enterococcus* [139, 158–160]. Membrane fluidity is determined by lipid head group packing and fatty acid disorder in the membrane. It is strongly influenced by temperature but is also affected by the properties of the fatty acids that compose the phospholipids [161, 162]. For example, due to the lack of a double bond, saturated fatty acids are straight-chained and so can pack together more closely than equivalent unsaturated fatty acids, resulting in less fluid membranes [163]. Similarly, branched-chain fatty acids result in more fluid membranes than equivalent straight chain fatty acids [161, 163, 164]. The amount of staphyloxanthin present in the membrane also affects the fluidity of *S. aureus* membranes, as its polar nature adds order to the membrane, decreasing its fluidity [161, 165].

While many studies have observed that strains with reduced daptomycin susceptibility have altered fluidity compared to susceptible strains, the exact role that membrane fluidity plays is unclear. *In vitro* passage of *S. aureus* strains in sub-lethal daptomycin concentrations led to increased abundance of the antioxidant carotenoid membrane pigment staphyloxanthin, which led to decreased membrane fluidity [139, 158, 166]. By contrast, analysis of daptomycin susceptible/non-susceptible strain pairs revealed increased membrane fluidity in *S. aureus* DNS strains and decreased fluidity in daptomycin-resistant enterococcal strains [159, 160, 167].

## Non-susceptibility due to changes in the bacterial cell wall

**Fig. 5.**
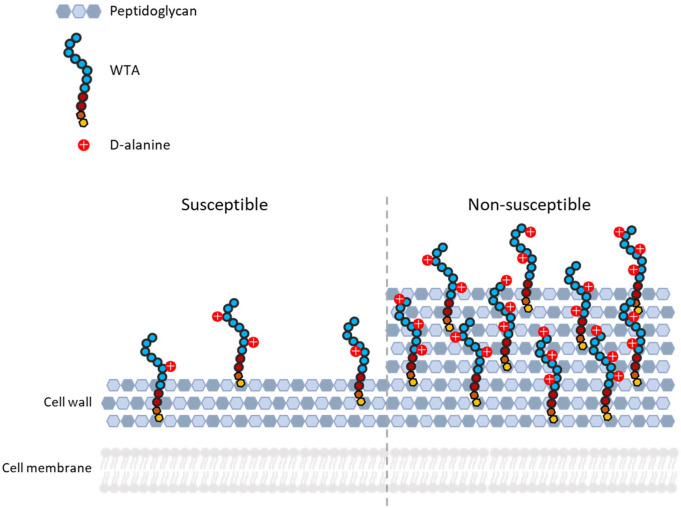
Cell wall of daptomycin-susceptible (left) and non-susceptible (right) *S. aureus *. Daptomycin non-susceptible strains have been observed to have increased levels of d-alanylated wall teichoic acid (WTA) and thicker cell walls than susceptible strains.

For daptomycin to reach its membrane-localized target it must pass through the cell wall, a 20–40 nm thick layer composed mainly of peptidoglycan and teichoic acids [168]. Changes in the cell wall, which reduce the ability of daptomycin to penetrate this layer, can therefore mediate DNS [134] (Fig. 5).

One important factor which affects how easily daptomycin can pass through the cell wall is the extent to which teichoic acids are modified with d-alanine, a process that is mediated by the products of the *dltABCD* operon [134]. The cell wall is negatively charged due to the high phosphate content of the teichoic acids. However, modification of teichoic acids with positively charged d-alanine groups reduces the net negative charge [169]. Transcriptomic analysis has revealed that overexpression of the *dltABCD* operon is frequently observed in DNS isolates [139, 145, 170–172] and may also contribute to a loss of daptomycin susceptibility in enterococci [157]. Similarly to the increase in LPG, the resulting increase in the positive charge of the cell wall is thought to contribute to repulsion of daptomycin [173–175]. Moreover, in addition to repelling daptomycin, it has been suggested that the increased charge also causes the teichoic acids to repel each other, leading to a more rigidlystructured wall which is harder for daptomycin to penetrate [176].

It has been observed that as many as 80 % of vancomycin intermediate *S. aureus* (VISA) strains show reduced daptomycin susceptibility, despite not having been exposed directly to the lipopeptide antibiotic [177, 178]. The characteristic phenotype of these VISA strains is a thickened cell wall, suggesting that this also protects against daptomycin [177, 179–181]. Similarly, daptomycin resistance in clinical enterococci isolates has also been linked to increased cell wall thickness, suggesting a broadly conserved mechanism [157, 160]. In agreement with this, serial passage in daptomycin led to increased cell wall thickness, and several studies comparing daptomycin susceptible and non-susceptible strain pairs have identified thicker cell walls in the less susceptible isolates [158, 159, 174, 175, 182, 183].

Transcriptomic analysis of these strain pairs has revealed upregulation of genes not only involved in peptidoglycan synthesis, but also in WTA synthesis [175]. Increased WTA levels make the wall denser and provide more potential sites for d-alanylation leading to increased daptomycin repulsion. The exact mechanisms by which these changes in the cell wall occur are not known, but they are thought to be mediated by VraSR and WalKR, regulators of the cell wall stress response and cell wall synthesis respectively, which are commonly upregulated in DNS and VISA strains [145, 184].

DNS has also been associated with polymorphisms in *rpoB* and *rpoC*, which encode subunits of RNA polymerase [138, 146]. However, the basis of the reduced susceptibility of these mutants is thought to be altered expression of *dltABCD* or increased cell wall thickness [185, 186].

## Daptomycin tolerance

Whilst DNS can result in treatment failure, it does not explain all or even most cases. More than 99.9 % of infections are caused by bacteria that are classed as daptomycin susceptible by MIC testing [187]. Additionally, while there are reports of resistance developing during treatment [188, 189], this is rare, and in many cases daptomycin treatment fails despite the infection being caused by a bacterium with an MIC classed as susceptible [190–192].

An alternative explanation for treatment failure is the presence of antibiotic-tolerant bacteria at infection sites. The concept of antibiotic tolerance was first described in 1944 and since then it has been implicated in many chronic and relapsing infections [193]. Tolerant bacteria are able to survive exposure to a normally lethal antibiotic concentration, despite not showing increased MIC values [194]. In contrast to resistance, tolerant bacteria are unable to replicate in the presence of the antibiotic but instead show a slower rate of killing than susceptible bacteria, only resuming growth when the antibiotic is removed. Tolerance can be genetic, due to the presence of heritable mutations, or can be phenotypic and non-inheritable. Phenotypic tolerance can apply to the whole bacterial population, or to a sub-population, where it is termed persistence [194].

Very few studies have investigated daptomycin tolerance and therefore little is known about the mechanisms responsible or whether it plays a role in treatment failure. The best-characterized daptomycin-tolerant mutant is the *S. aureus pitA6* mutant, which was generated via serial daptomycin exposure and which was found to contain a point mutation in the putative phosphate transporter *pitA* [195, 196]. Transcriptomics revealed a large number of differentially regulated genes between the mutant and its parental strain, but the tolerance of this mutant was ascribed to an upregulation of the *dltABCD* operon induced by the accumulation of intracellular phosphate [195, 196].

A second *in vitro* serial daptomycin exposure experiment with *S. aureus* resulted in the generation of several additional daptomycin-tolerant mutants, identifying new loci with potential roles in tolerance, including *hmp1, rimP, rsh, map1* and *amaP* [197]. None of these observed polymorphisms were predicted to affect the cell membrane, and the mechanisms by which polymorphisms in these genes resulted in tolerance were not determined [197]. Similarly, null mutations in *asp23* and *dsp1* have also been reported to result in tolerance to daptomycin, vancomycin and cationic AMPs [198]. The mechanisms of tolerance of these mutants were unknown, but they were suggested to be due to changes in the cell membrane [198].

Therefore, there is evidence that daptomycin tolerance occurs *in vitro,* although whether it contributes to treatment failure is unknown, in part because standard diagnostic assays are unable to detect tolerance.

## Inactivation of polymyxins and daptomycin via membrane decoys

In addition to changes in the cell envelope, there is evidence that bacteria can survive exposure to polymyxins and daptomycin by releasing the targets of these antibiotics into the extracellular space [199–204]. These act as decoys by binding the antibiotics and thus protecting the bacterial cell membranes. In the case of polymyxins, there is evidence that *P. aeruginosa* releases large quantities of LPS during exposure to the antibiotics [202]. Whilst this is probably a consequence of membrane damage caused by the polymyxins, the free LPS binds the antibiotics and enables bacteria that survived initial exposure to the antibiotic to replicate and thus restore the population [202]. Furthermore, release of outer membrane vesicles can sequester colistin [203].

Similarly, several Gram-positive pathogens, including *S. aureus *, *Enterococcus faecalis* and pathogenic streptococci, have been shown to release PG in response to daptomycin exposure, which protects the bacteria from the lipopeptide [200, 201]. This process appears to require phospholipid biosynthesis, since the presence of the fatty acid synthesis (FASII) inhibitor AFN-1252 blocks daptomycin-induced phospholipid release, suggesting it is not simply a consequence of daptomycin-mediated membrane damage [205]. Furthermore, daptomycin-induced phospholipid release does not occur in all strains of *S. aureus *, which may provide a route to a greater mechanistic understanding of this phenomenon [205, 206].

The phenomenon of decoys is part of a growing recognition that bacteria release membrane components into the extracellular space that can detoxify antibiotics and host defences, although the clinical relevance of decoy production is currently unclear [199]. In the context of the cystic fibrosis lung, there is abundant free LPS which could sequester polymyxins [199]. However, it remains to be determined whether this affects treatment efficacy. Similarly, whilst there is evidence that phospholipid release promotes *S. aureus* survival during daptomycin exposure *in vivo*, it remains to be determined whether this phenomenon contributes to daptomycin treatment failure in humans [199].

## Polymyxins and daptomycin synergize with several other antibiotics, but this does not improve patient outcomes

One approach that has been taken to improve treatment outcomes with daptomycin or polymyxin antibiotics is to use them in combination with other antibiotics. This is supported by *in vitro* assays showing that both classes of antibiotic synergize with several other antibiotics [207, 208].

In the case of polymyxins, the disruption caused to the LPS monolayer removes a large permeability barrier and sensitizes bacteria to several antibiotics that would not normally be able to penetrate Gram-negative bacteria such as rifampicin and erythromycin, as well as several other antibiotics that can normally cross the outer membrane [124, 207]. Surprisingly, colistin also sensitizes polymyxin-resistant *E. coli* to hydrophobic antibiotics such as rifampicin, meaning that the bacterium is resistant to each drug separately, but susceptible to the two drugs in combination [37, 38].

There are relatively few clinical studies that have tested colistin in combination with rifampicin. However, two reasonably well-powered studies found that whilst the combination improved microbiological cure relative to colistin alone, this did not result in improved patient outcomes [209, 210]. Furthermore, although colistin synergizes with carbapenem antibiotics against most carbapenem-resistant bacteria, a randomized controlled trial did not support the combination of colistin with meropenem, with similarly high treatment failure rates in those who received both drugs (>50 %) compared to patients who received colistin monotherapy [13, 211].

There has been intense interest in combining daptomycin with beta-lactams, since these consistently show synergy *in vitro* [212]. However, whilst daptomycin combined with a beta-lactam improved bacterial eradication, it did not lead to improved patient outcomes [213].

Therefore, whilst both polymyxins and daptomycin synergize with other antibiotics, the results from clinical trials do not suggest that they improve patient survival. The reasons for this warrant investigation, particularly whether earlier use of combination therapy or sequential administration of antibiotics would enhance outcomes. In support of this second approach, a recent report showed improved outcomes for patients given a beta-lactam before they received vancomycin [214].

## New polymyxins and lipopeptides

One way to improve activity, reduce host toxicity and/or overcome resistance is to develop new analogues of existing antibiotics. The size and complexity of lipopeptide and polymyxin antibiotics makes this challenging. However, progress has been made in both the synthesis and determination of structure–activity relationships (SARs) of polymyxins [29, 215–217]. For example, a recent and very comprehensive analysis of polymyxin SARs, facilitated by the development of two total synthetic routes for polymyxins, identified polymyxin analogues with significantly enhanced activity and reduced host cell toxicity [29]. Intriguingly, some analogues also had activity against an intrinsically-resistant strain of *Moraxella *, which may indicate that it is possible to generate polymyxins with broad anti-Gram-negative activity, as well as activity against strains resistant to currently available polymyxins.

Progress in polymyxin synthesis and SARs has resulted in several new polymyxins entering preclinical testing, including *in vivo* toxicity studies (summarized in detail in the literature [217, 218]). Whilst results have been mixed, several analogues with improved activity and/or reduced toxicity have been identified, providing confidence that next-generation polymyxins will reach the clinic.

A total synthetic route for daptomycin has also been reported [219], as well as a chemoenzymatic approach [220], both of which have led to SAR data [221]. Whilst most analogues were less active than daptomycin against daptomycin-susceptible bacteria, it did reveal analogues with significantly improved activity against daptomycin-resistant strains of *S. aureus* and enterococci [222].

Furthermore, although daptomycin is the only lipopeptide antibiotic licensed for clinical use, a large number of related molecules with potent antibacterial activity have been identified [223]. This includes the malacidins, which were identified using computational approaches, demonstrating the potential of genome mining to identify new antibiotics without the need to culture bacteria, a major hurdle in the discovery of natural product antibiotics [224]. Interestingly, malacidin does not cause membrane damage but instead functions by interrupting cell wall biosynthesis by targeting lipid II, while another lipopeptide, friulimicin, inhibits cell wall biosynthesis by binding bactoprenol phosphate. Therefore, this class of antibiotics appears to have diverse mechanisms of action [225].

In addition to new polymyxins and lipopeptides, there is also interest in developing novel drug delivery systems to enhance efficacy, improve stability and reduce host toxicity [226–228].

## Future perspectives and remaining questions

The polymyxins and lipopeptide classes of antibiotics are often used as drugs of last resort, but they are less efficacious than frontline treatments and polymyxins are associated with host toxicity. Improving treatment outcomes is therefore a pressing concern but is hampered by a poor understanding of the mode of action and difficulties in defining and identifying resistance, especially since small differences in susceptibility can have large impacts on treatment outcomes. Furthermore, whilst *in vitro* studies indicate improved antibiotic activity when used in combination with other drugs, this is yet to translate into improved patient outcomes. As such, more work is needed to find ways to make these drugs of last resort as effective as possible and we propose five priority areas for future research efforts.

## Priority areas

1. Determine fully the mechanisms of action of both antibiotic classes.
2. Determine the mechanisms and clinical importance of colistin heteroresistance and daptomycin tolerance.
3. Determine how the host environment influences susceptibility to polymyxins and daptomycin, both as single agents and when used in combination therapy.
4. Determine how best to combine polymyxins and daptomycin with other drugs.
5. Identify and develop new polymyxins and lipopeptides with improved efficacy and reduced host toxicity.
